# Comparative analysis of socio-demographic and clinical profiles among suicide attempt patients: A three-month follow-up study

**DOI:** 10.6026/973206300222273

**Published:** 2026-04-30

**Authors:** Bhanpratap Ahirwar, Vinay Verma, Shivam Chaudhary, Sachin Parmar

**Affiliations:** 1 Departmentof General Medicine, V.K.S. Government Medical College, Neemuch, Madhya Pradesh, India; 2 Department of Community Medicine, V.K.S. Government Medical College, Neemuch, Madhya Pradesh, India

**Keywords:** Suicide attempt, socio-demographic profile, mental health disorders

## Abstract

Suicide is a significant global health issue, adversely affecting individuals, families, and society at large. This cross-sectional research involved 62 suicide attempt survivors in 12 months of the study who had been hospitalized at the MGM Medical College and MY Hospital, Indore, India with a 3 months follow up. Psychiatric examination involved the use of Columbia-Suicide Severity Rating Scale (C-SSRS) at the time of admission and follow-up check. Major participants were young adults (18-30 years) with a margin of females over males. The most frequent methods of attempt were poisoning and overdosing of drugs and the most prevalent diagnosis was depression. At 3 months, 38 had reported improved health and 42 had endured therapy. We found strong association between family support and better outcomes and continued treatment attendance.

## Background:

Suicide attempts represent a major global public health challenge, requiring the detailed profiling of patients across demographic and clinical domains to guide targeted prevention and structured aftercare [1]. The burden of suicide is disproportionately high in low- and middle-income countries, where limited mental health infrastructure often leaves emergency departments (EDs) as the primary point of contact for individuals in crisis. Emergency and inpatient services in India frequently manage suicide attempt survivors, creating a critical opportunity to characterize local patterns and link them to follow-up outcomes [2]. Understanding the specific demographic and clinical correlates of suicide attempts is essential for developing effective prevention strategies. Suicide attempt cohorts from Indian tertiary-care settings consistently report substantial psychiatric morbidity, highlighting the need for comprehensive clinical assessment immediately following medical stabilization [3]. Research indicates that adolescent and young adult samples in emergency departments exhibit distinct demographic and clinical patterns that warrant age-sensitive interventions [4]. For instance, the psychosocial stressors affecting a young adult aged 18-30 such as academic pressure, unemployment and relationship instability-differ significantly from those affecting older populations, necessitating tailored approaches to risk assessment and care [5]. Studies of emergency-room presentations for suicidal behavior emphasize the relevance of the social context and clinical correlates when evaluating patients presenting after an attempt [6]. Self-poisoning remains a common mechanism of attempt in many settings and trend data show large burdens of suicidal self-poisoning among poison-center admissions with identifiable demographic and psychosocial correlates [7]. In India, pesticide self-poisoning has been a key contributor to suicide mortality and regulatory actions on highly hazardous pesticides have been examined for their potential association with suicide trends [8]. The high prevalence of pesticide ingestion in agricultural regions contrasts with drug overdoses in urban settings, reflecting the accessibility of lethal means as a critical determinant of attempt outcomes [7]. Standardized measurement improves comparability and clinical decision-making in suicidology research and practice. The Columbia-Suicide Severity Rating Scale (C-SSRS) and related approaches have been evaluated in emergency department populations, supporting structured assessment of suicidal ideation and behavior [9]. The weeks and months after a suicide attempt represent a high-risk period for recurrence, making early continuity of care an essential outcome domain [10]. A systematic review and meta-analysis has recommended active contact and follow-up interventions for suicidal patients discharged from emergency departments to reduce repeat attempts, particularly within the first six months [11]. Digital and brief-contact approaches, including mHealth-oriented models and SMS-based interventions, have been explored as scalable strategies to maintain engagement after discharge [12]. Randomized controlled trial evidence is emerging for SMS-based brief contact interventions following self-harm/suicide-related presentations [10]. Involving families and carers is increasingly recognized as relevant to aftercare, particularly for adolescents and young people discharged after suicidal crises [13]. Therefore, it is of interest to prospectively analyze the socio-demographic and clinical profiles of suicide attempt survivors in a tertiary care setting to identify specific modifiable risk factors and evaluate the efficacy of short-term follow-up in improving post-discharge outcomes.

## Materials and Methods:

This was a cross-sectional study conducted to assess the sociodemographic and clinical profile of patients who attempted suicide, followed by a 3-month follow-up. The study was conducted in the Department of Medicine, MGM Medical College and Maharaja Yashwant Rao Hospital, Indore (Madhya Pradesh), during a period of 12 months, from September 2019 to August 2020. The study adhered to the guidelines and regulations of the Institutional Ethics Committee.

## Sample size:

A total of 62 patients were included in the study, whose data were collected during their admission to the Medical Intensive Care Unit (MICU) and the general ward of the hospital.

## Inclusion criteria:

The following criteria were used for inclusion in the study:

[1] All patients who were admitted to the Medicine ICU or general ward of M.Y. Hospital, Indore, with a history of suicide attempt

[2] Patients aged 18 years and above

[3] Patients who provided valid informed consent for participation

[4] In cases where patients were unable to give consent (due to severe condition), informed consent was obtained from their legal representative/guardian.

## Exclusion criteria:

The following groups of patients were excluded from the study:

[1] Prisoners

[2] Pregnant females

[3] Patients who did not provide valid informed consent

[4] Terminally ill patients who were not expected to survive for more than 28 days from the time of participation.

## Diagnostic criteria:

The diagnosis of a suicide attempt was established based on the following criteria:

## History:

Information was gathered from the patient or their caregivers regarding the method, timing and circumstances of the suicide attempt.

## Physical examination:

A thorough physical examination was performed to identify any injuries or complications resulting from the suicide attempt.

## Laboratory and radiological investigations:

Relevant investigations (*e.g.*, blood tests, imaging) were carried out to assess the extent of any self-inflicted injuries and complications.

## Columbia-suicide severity rating scale (C-SSRS):

The C-SSRS was used to assess the severity of suicide ideation and behaviors, providing a standardized measure of the patient's suicidal tendencies.

## Mental status examination (MSE):

An MSE was conducted to evaluate the patient's psychological state, cognition and any underlying psychiatric conditions that may have contributed to the suicide attempt.

## Study protocol:

## Data collection:

Upon admission, each patient was evaluated for sociodemographic data (age, gender, education level, occupation, marital status, etc.,) and clinical factors (mental health history, substance abuse, family history, etc.).

## Follow-up:

After the initial assessment and treatment, all patients were followed up for three months through regular outpatient visits or telephonic assessments.

During follow-up, the patients were evaluated for:

[1] Changes in suicidal ideation or attempts.

[2] Psychiatric evaluation for ongoing depression, anxiety or other mental health disorders.

[3] Family and social support.

[4] Compliance with prescribed treatments and medications.

## Results and Discussion:

A total of sixty-two patients including thirty males and thirty-two females were enrolled in the study which had a predominantly young adult's population. Age group 18-30 years was the most common age group that constituted thirty-eight patients in the study. Most of the patients had higher education (n=35) and were working (n=33). Family support was present in thirty-eight patients (Table 1 - see PDF). As for the clinical profile, poisoning and overdose were the most frequent modes of suicide action (n = 22 each), almost half of the patients (n = 26) had a previous suicide attempt, depression was the most common comorbid mental health condition (n = 38), whereas alcohol misuse was the most common form of substance misuse (n = 22) (Table 2 - see PDF). At the three month follow up, majority reported health improvement (n=38) and were in current therapy (n=42). Moreover, family support increased significantly after the index attempt (n=53) (Table 3 - see PDF). The study conducted a multi-center evaluation of 108 self-harm patients, which reported important clinical viewpoints along with risk factor analysis, the outcomes. "The most common reason to attempt self-harm was financial problems (31 cases). The second major reason was relationship problems (26 cases). The third most common reason was chronic illness (22 cases)". After evaluation, the most common outcome was regular follow-up visits (38 cases). The other outcome was improvement in mental health (42 cases). The study recognizes the need for care after an attempt of self-harm (Table 4 - see PDF). The study included 62 suicide-attempt patients (30 males, 32 females). The majority of attempters were aged 18-30 years (61.3%, n=38), with a slight female predominance in this age group (20 females versus 18 males). Marital status was split between single (25/62) and married (27/62) individuals (Table 1 - see PDF). This demographic profile aligns with international and regional literature, which identifies young adulthood as a period of peak vulnerability [1]. Singh *et al.* whose study reported a female predominance of 66% compared to the slight female majority (51.6%) observed in the current study, found 48% of attempters to be married [14]. Tang *et al.* done an Emergency Department based study (2026), mean age 26.25 years, 52.3% female) compared the effectiveness of interventions based on psychological care in which the researcher found that re-suicide rates were significantly lower (11.45% versus 24.07) in the intervention group at a one-month follow-up [15]. Rajnish *et al.* (2024) study assessed predictors of suicidal behavior in young adults (18-35 years) across 18 months at a north Indian tertiary care hospital [16]. The transition from adolescence to adulthood involves significant psychosocial stressors, including educational pressure and relationship instability, which are amplified in developing economies. Interestingly, 56.5% of patients (35/62) possessed higher education. This corresponds with literature showing that approximately 63% of suicide attempters have completed matriculation or higher education [5]. This finding challenges the traditional view of low education as a primary risk factor and may reflect the "unemployment of the educated" in the Indian context, where status incongruence creates severe psychological distress. Clinically, poisoning (22/62) and overdose (22/62) were the most common modes of attempt, followed by hanging (18/62).

The slight female predominance (51.6%) observed in the study aligns with the "gender paradox" in suicidology, where females exhibit higher rates of attempts while males demonstrate higher rates of completed suicide [17]. In our cohort, poisoning and overdose combined accounted for 71% of attempts (Table 2 - see PDF). Research consistently shows that females prefer these less lethal methods, whereas males more frequently resort to highly lethal means such as hanging [18, 19] the high incidence of poisoning (35.5%) points to the continued relevance of means restriction strategies, such as regulating access to hazardous pesticides, which has proven effective in agrarian settings [20]. Psychiatrically, depression was the pre-dominant diagnosis (61.3%, 38/62), followed by anxiety (30.6%, 19/62). This reinforces depression as the single most significant clinical risk factor, consistent with studies indicating that over 90% of serious suicide attempters have a mental disorder at the time of attempt [21]. Substance abuse was present in 64.5% of cases, with alcohol use more frequent among males (12 versus 10) and drug use slightly higher among females (10 versus 8) (Table 4 - see PDF). This reflects established patterns where substance use disorders increase suicide risk through disinhibition [22]. Key reported risk factors included financial problems (50%, 31/62), family history of suicide (29%, 18/62) and relationship issues (41.9%, 26/62). The high prevalence of financial stress is critical; recent epidemiological evidence suggests that cumulative financial strain encompassing debt and unemployment can increase the risk of a suicide attempt by up to 20-fold [20]. Furthermore, the 29% prevalence of family history aligns with research identifying familial transmission as a significant risk factor, with studies indicating that a family history of suicide attempts increases risk by nearly 6-fold [23]. At the 3-month follow-up, 61.3% of patients (38/62) reported improved health and 67.7% (42/62) continued with therapy. Notably, reported family support increased from 61.3% at baseline to 85.5% (53/62) post-attempt (Table 3 - see PDF). This positive correlation between family support and favorable outcomes contrasts with reports of family disruption following attempts [24]. Suggest effective mobilization of social resources in this cohort. The high rate of regular follow-up visits (61.3%) compares favorably to typical dropout rates [11]. This is encouraging, as research demonstrates that early and consistent follow-up care is essential for reducing recurrence risk [9, 10].

## Conclusion:

We show that the study findings largely align with known data on suicide attempt profiles, with some notable variations in gender distribution and follow-up outcomes. The data support the multifactorial nature of suicide risk, involving demographic, clinical and psychosocial factors. Thus, the positive three-month outcomes emphasize the importance of structured follow-up care and family involvement in suicide prevention strategies.
